# Mixtures of natural antimicrobials can reduce *Campylobacter jejuni*, *Salmonella enterica* and *Clostridium perfringens* infections and cellular inflammatory response in MDCK cells

**DOI:** 10.1186/s13099-021-00433-5

**Published:** 2021-06-07

**Authors:** Igori Balta, Adela Marcu, Mark Linton, Carmel Kelly, Ozan Gundogdu, Lavinia Stef, Ioan Pet, Patrick Ward, Myriam Deshaies, Todd Callaway, Phittawat Sopharat, Gratiela Gradisteanu-Pircalabioru, Nicolae Corcionivoschi

**Affiliations:** 1 Food Microbiology, Bacteriology Branch, Veterinary Sciences Division, Agri-Food and Biosciences Institute, 18a Newforge Lane, Belfast, BT9 5PX Northern Ireland UK; 2 Faculty of Animal Science and Biotechnologies, University of Agricultural Sciences and Veterinary Medicine, 400372 Cluj-Napoca, Romania; 3 Faculty of Bioengineering of Animal Resources, Banat University of Agricultural Sciences and Veterinary Medicine-King Michael I of Romania, 300645 Timisoara, Romania; 4 Department of Infection Biology, Faculty of Infectious & Tropical Diseases, London School of Hygiene & Tropical Medicine, Keppel Street, WC1E 7HT, London, UK; 5 Auranta, Nova UCD, Belfield, Dublin, Ireland; 6 Department of Animal and Dairy Science, University of Georgia, Athens, GA USA; 7 Research Institute of University of Bucharest (ICUB), 300645 Bucharest, Romania

**Keywords:** *C. jejuni*, *S. enterica*, *C. perfringens*, Natural antimicrobials, Hydrogen peroxide, ERK kinase, MDCK cells, Virulence

## Abstract

**Background:**

The classification of natural antimicrobials as potential antibiotic replacements is still hampered by the absence of clear biological mechanisms behind their mode of action. This study investigated the mechanisms underlying the anti-bacterial effect of a mixture of natural antimicrobials (maltodextrin, citric acid, sodium citrate, malic acid, citrus extract and olive extract) against *Campylobacter jejuni* RC039, *Salmonella enterica* SE 10/72 and *Clostridium perfringens* ATCC® 13124 invasion of Madin–Darby Canine Kidney cells (MDCK).

**Results:**

Minimum sub-inhibitory concentrations were determined for *Campylobacter jejuni* (0.25%), *Salmonella enterica* (0.50%) and *Clostridium perfringens* (0.50%) required for the in vitro infection assays with MDCK cells. The antimicrobial mixture significantly reduced the virulence of all three pathogens towards MDCK cells and restored the integrity of cellular tight junctions through increased transepithelial resistance (TEER) and higher expression levels of ZO-1 (zonula occludens 1) and occludin. This study also identified the ERK (external regulated kinase) signalling pathway as a key mechanism in blocking the pro-inflammatory cytokine production (IL-1β, IL-6, IL-8, TNF-α) in infected cells. The reduction in hydrogen peroxide (H_2_O_2_) production and release by infected MDCK cells, in the presence of the antimicrobial mixture, was also associated with less tetrathionate formed by oxidation of thiosulphate (p < 0.0001).

**Conclusion:**

The present study describes for the first time that mixtures of natural antimicrobials can prevent the formation of substrates used by bacterial pathogens to grow and survive in anaerobic environments (e.g. tetrathionate). Moreover, we provide further insights into pathogen invasion mechanisms through restoration of cellular structures and describe their ability to block the ERK–MAPK kinase pathway responsible for inflammatory cytokine release

## Background

Food-borne bacterial illnesses affect approximately 226 million people each year [1, 2]. Infections caused by food-borne zoonotic agents, such as *Campylobacter* spp., *Salmonella* spp., and *Clostridium* spp., are associated with high morbidity rates worldwide in both humans and animals [3]. Household pets can also contract these pathogens through non-food sources, such as the environment, pet feed and contact with infected animals or humans [4].

Poultry meat is considered the main source of *Campylobacter* for human infections, however, the presence of this bacterium in dogs and cats increases significant public health concerns as pets can potentially be considered a transmission vector [5]. In dogs, *Campylobacter* spp. infection can trigger acute polyradiculoneuritis (APN), a canine version of Guillen-Barre Syndrome (GBS) which displays similar pathologies [6]. In companion animals, salmonellosis is mainly caused by *Salmonella enterica* serovar Typhimurium and specifically, in dogs is transmitted via the consumption of raw feeds rather than processed feeds [7]. Following infection, the bacterium is primarily isolated from the mesenteric lymph nodes and intestinal tracts. Dogs can be asymptomatic carriers for more than 42 days, during which time the pathogen is easily transmissible to humans [8]. *Clostridium perfringens* is mainly associated with acute haemorrhagic diarrhoea syndrome (AHDS) in dogs [9]. Symptoms normally are a mild or self-limiting diarrhoea, but in some cases can also lead to fatal acute haemorrhagic diarrhoea. In cases of severe haemorrhagic canine gastroenteritis,a severe necrotising inflammation of the intestinal tract epithelium can occur which can lead to rapid mortality [10].

Mixtures of natural antimicrobials (e.g. plant extracts or organic acids) have been shown to prevent colonisation or infection of the epithelium in a variety of hosts, including ruminants, monogastrics and aquatic crustaceans [11–16]. The anti-infective mode of action occurs by improving the host gastrointestinal epithelium integrity and downregulating some of the vital bacterial virulence factors (e.g. motility, bacterial polysaccharides). At the host level, the antimicrobial mode of action was related to the restoration of tight junction (TJ) integrity, and diminished inflammation via a reduction in pro-inflammatory cytokine secretion throughout the gut [15]. Common canine pathogens (e.g., *Campylobacter* spp., *Salmonella* spp., and *Clostridium* spp.) are inhibited by mixtures of these natural antimicrobials [17, 18].

Understanding the biological mechanism by which natural antimicrobials reduce bacterial infections and reduce epithelial inflammation is essential to establish best practices for end users, and ensure that their specific anti-bacterial effect can be consistently utilized. Bacterial pathogens, including *Campylobacter*, can disrupt epithelial tight junctions (TJ) upon infection [19] and will stimulate the host NADPH oxidases to produce and release extra and intra-cellular hydrogen peroxide (H_2_O_2_) [20]. At the cytoplasmic level, consequences of H_2_O_2_ production include the activation of the extracellular signal-regulated kinase (ERK), which is central to the signalling cascade leading to pro-inflammatory cytokine production [21, 22]. The involvement of the ERK pathways in pro-inflammatory events was previously described [23] as being closely related to bacterial infection [24]. Other natural antimicrobial extracts (e.g., ambuic acid) have an anti-inflammatory action that blocks activation of the ERK signalling pathway in lipopolysaccharide (LPS)-treated cells [25].

Natural antimicrobials can serve as both treatment and prophylaxis by preventing a drop in trans-epithelial resistance and restoring cellular TJ integrity [26]. The present study investigated the possible role of natural antimicrobials and their anti-pathogenic effect against *C. jejuni* RC039, *S. enterica* SE 10/72 and *C. perfringens* ATCC® 13124 in canine derived Madin–Darby Canine Kidney cells (MDCK). Further, we hypothesized that natural antimicrobials could prevent bacterially-induced oxidative stress and restore cellular structure integrity (including TJ or ZO-1 and occludin) damaged as a result of cytoplasmic H_2_O_2_ release, and could block ERK activation and prevent inflammation and pro-inflammatory cytokine release. It is clear now that attenuation of ROS induced oxidative stress induced by the antimicrobial mixture also led to a decrease in host-induced molecules (e.g. tetrathionate) a well-known electron acceptor used by bacterial pathogens to survive in oxygen depleted environments [27].

## Results

### Establishing the sub-inhibitory concentrations

**Fig. 1:**
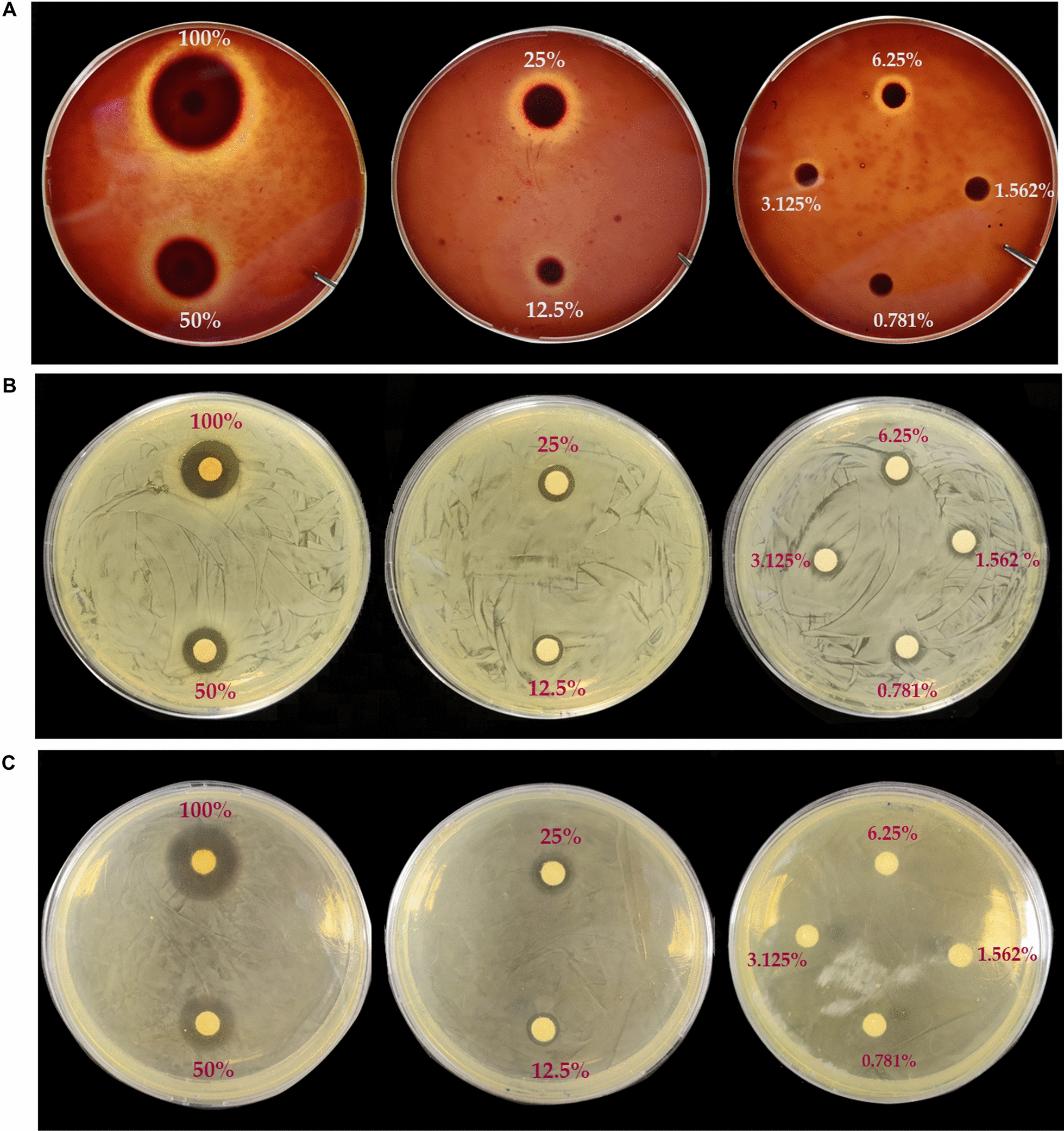
Representative Petri plate images showing the inhibitory zones at different concentrations of Auraguard against *C. jejuni* (**A**), *S. enterica* (**B**) and *C. perfringens* (**C**)

First we aimed to establish the anti-pathogenic effect of a natural antimicrobial mixture (Auraguard) and to determine the sub-inhibitory concentrations for *C. jejuni*, *S. enterica* and *C. perfringens*. Strong antimicrobial activity was detected against all three species (Fig. 1). The minimum inhibitory concentrations were 0.25% for *C. jejuni*, and 0.50% for *S. enterica* and *C. perfringens*. The minimum bactericidal concentrations were noted at 0.50% for *C. jejuni*, at 1% for *S. enterica* and *C. perfringens* (Table 1). Based on these results we used concentrations of 0.25% for *C. jejuni*, and 0.5% for *S. enterica* and *C. perfringens* to test their inhibitory effect in preventing the infection of MDCK cells.

**Table 1:** Minimum inhibitory concentration (MIC) and minimum bactericidal concentration (MBC) activity of the antimicrobial mixture

Strains	MIC (%)	MBC (%)
*Campylobacter jejuni* (RC039)	0.25	0.50
*Salmonella enterica* (SE10/72)	0.50	1
*Clostridium perfringens* (ATCC® 13124™)	0.50	1

### Auraguard prevents the in vitro infection of MDCK cells by *C. jejuni*, *S. enterica* and *C. perfringens*

**Fig. 2:**
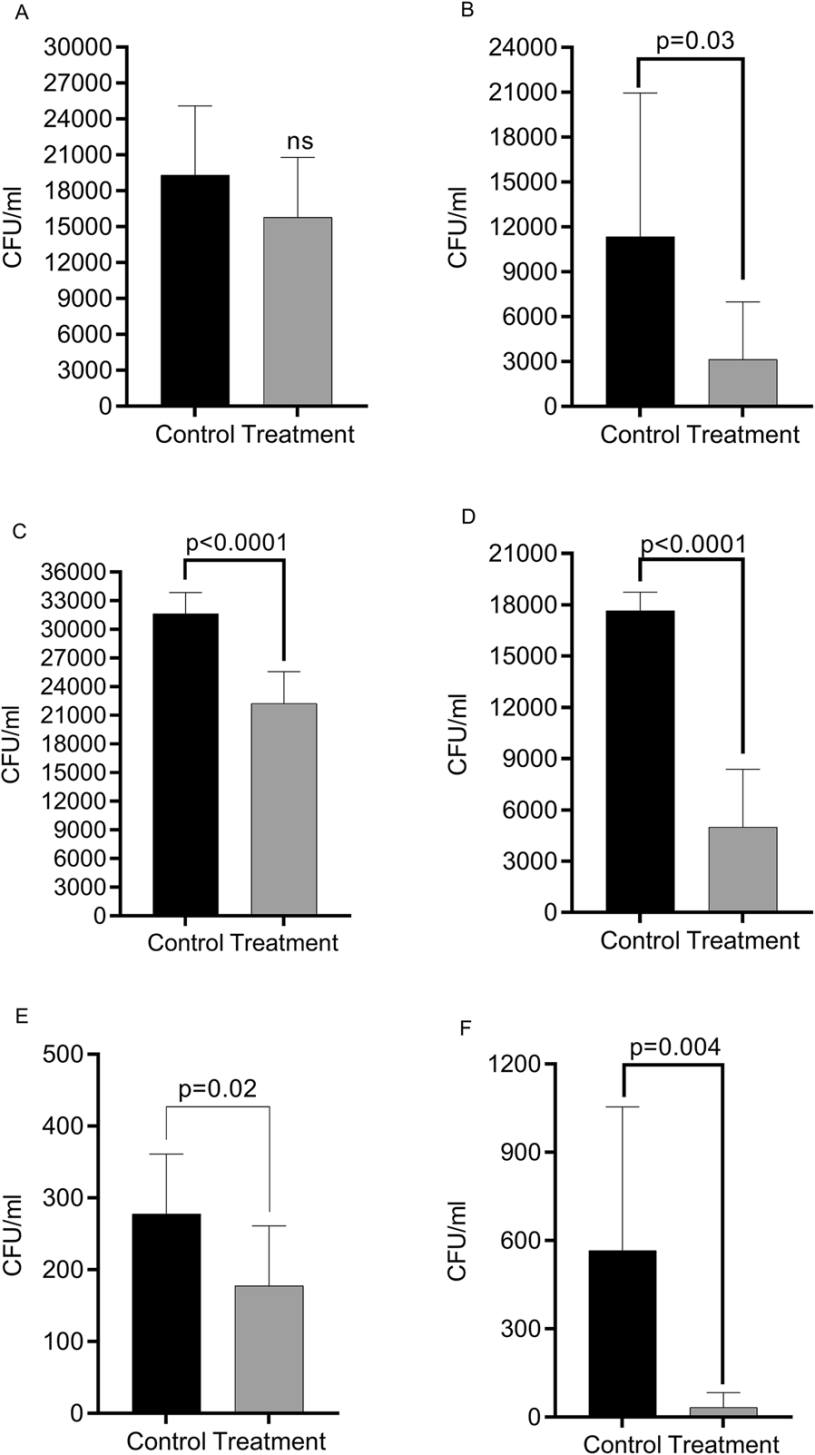
In vitro adhesion and invasion of Auraguard pre-treated (0.25% and 0.50%) MDCK cells by *C. jejuni* RC039 (**A**—adhesion, **B**—invasion), *S. enterica* (**C**—adhesion, **D**—invasion) and *C. perfringens* (**E**—adhesion, **F**—invasion). Results are expressed as CFU/ml. Error bars represent the standard deviation of means from three different experiments, each containing triplicate samples

In vitro infection assays were performed to identify if there was a direct effect on cellular invasion by Auraguard added at 0.25% and 0.50%. Exposure of MDCK cells prior to infection reduced (p = 0.03) the invasion of *C. jejuni* at concentrations of only 0.25% (Fig. 2B) with no significant decrease in adhesion (Fig. 2A). In the case of *S. enterica* there was a impact on both adhesion (Fig. 2C, p < 0.0001) and invasion (Fig. 2D, p = 0.0001) after pre-treatment of MDCK cells with 0.50% Auraguard. A similar pattern was observed in *C. perfringens* infections with impacts on both bacterial adhesion (Fig. 2E, p = 0.02) and cell invasion (Fig. 2F, p = 0.004), when 0.50% Auraguard was used to pre-treat the epithelial cells. Overall, results suggest that Auraguard prevented the adhesion and invasion of MDCK cells potentially by restoring the integrity of the cellular structures damaged by bacterial infection.

### The effect of Auraguard on paracellular permeability, TEER, ZO-1 and occludin expression in infected MDCK cells

**Fig. 3:**
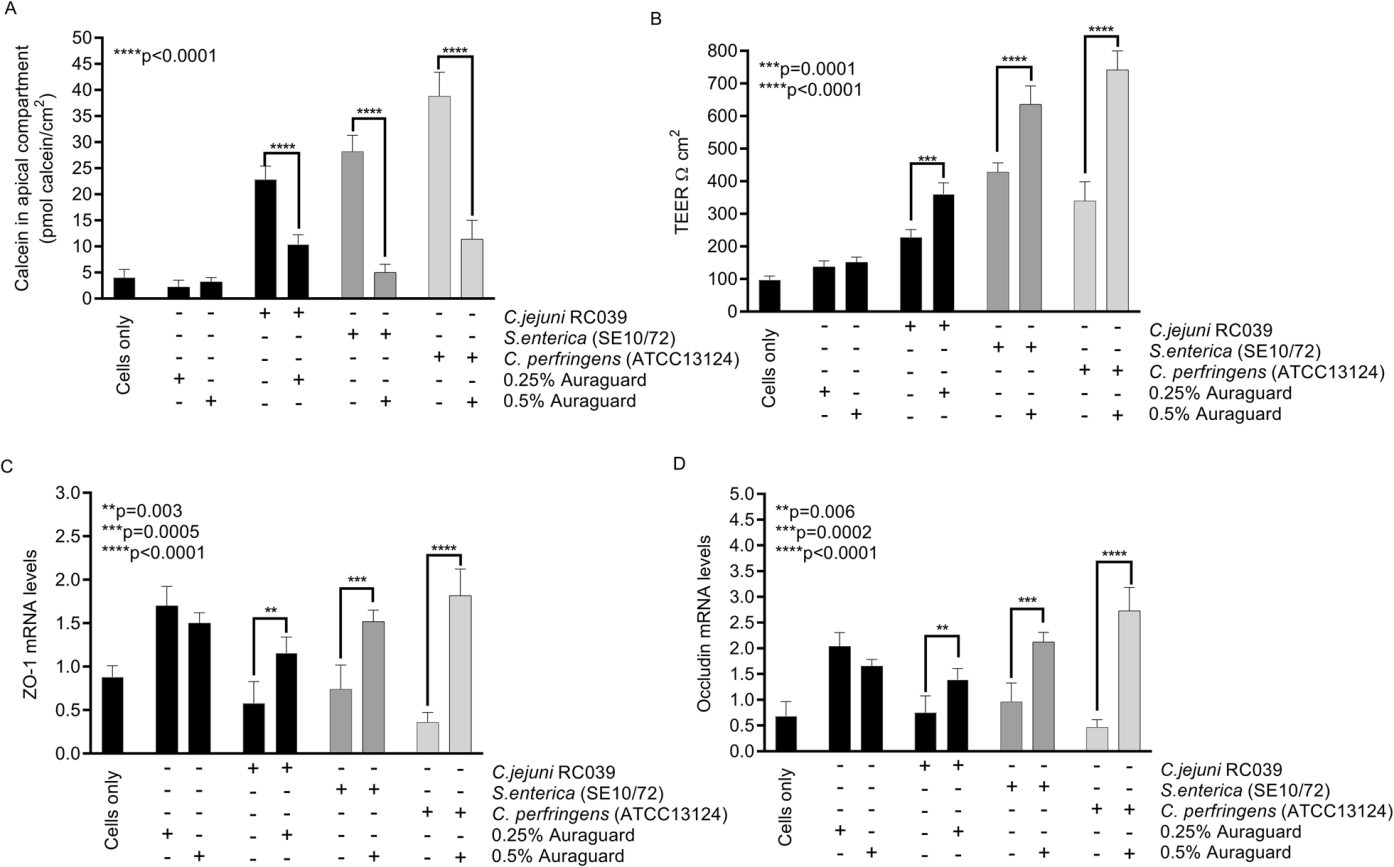
The effect of Auraguard on infected MDCK cells paracellular membranes calcein flux (**A**), TEER (**B**), ZO-1 (**C**) and occluding expression (**D**) during infection with *C. jejuni*, *S. enterica* or *C. perfringens*. Data are presented as means (SD) of triplicate independent samples and experiments

Following the in vitro infection assay results we investigated the impact of Auraguard on cell membrane restoration leading to reduced infectivity. Treatment of MDCK cells with 0.25% and 0.5% Auraguard decreased the paracellular calcein flux indicating improvements (****p < 0.0001) in cellular membrane integrity (Fig. 3A). We expected that an improvement in membrane permeability should be accompanied by an improvement of cellular tight junctions quantified by measuring the trans-epithelial resistance. Our results show that infection of MDCK cells with *C. jejuni, S. enterica* and *C. perfringens* decreased the trans-epithelial resistance of cell monolayers at 3 h post-infection (Fig. 3B). In contrast, Auraguard exposure of cells led to increased TEER values reducing the detrimental effect of pathogen infection (p < 0.0001). To further prove that Auraguard has a beneficial impact on cellular membrane structures we measured the effect on the mRNA levels of ZO-1 (Fig. 3C) and occludin (Fig. 3D). Results demonstrate that following infection with *C. jejuni* the addition of 0.25% Auraguard increased the expression of ZO-1 (p = 0.003) and occludin (p = 0.006) in cells. Similarly, an increase in ZO-1 and occluding expression (p = 0.0005 and p = 0.0002, respectively) occurred when MDCK cells were infected with *S. enterica* and exposed to 0.5% Auraguard*.* Infections with *C. perfringens* of MDCK cells pre-treated with 0.5% Auraguard also led to a significant increase in ZO-1 expression (p < 0.0001) and occludin (p < 0.0001). Collectively, these results suggest that Auraguard has a positive and restorative impact on cellular membrane integrity and prevents infection of epithelial cells.

### Auraguard blocks the ERK signal transduction pathway, prevents inflammation and the intra and extracellular H_2_O_2_ release in in MDCK cells but not bacterial invasion

**Fig. 4:**
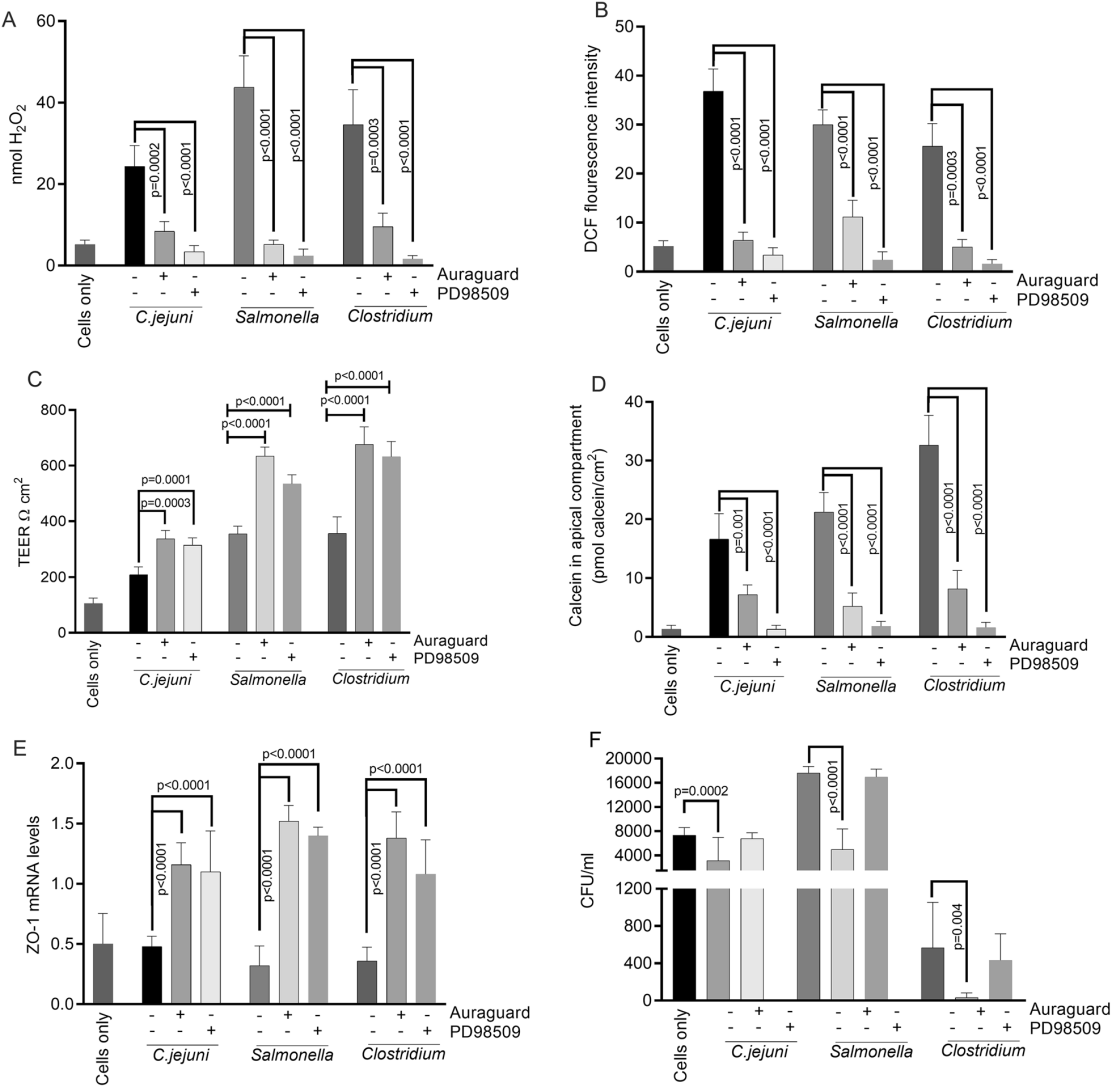
The effect of Auraguard and the ERK inhibitor PD98059 on infected MDCK cells. The extracellular levels of H_2_O_2_ released by the infected MDCK cells and Auraguard and PD98509 treated MDCK cells are presented in panel A and the intra-cellular levels in panel B. Panel C shows the effect on MDCK cells TEER with panel D describing the effect on paracellular permeability and in panel E the ZO-1 expression levels. The bacterial infectivity levels are shown in panel F. The significance levels are indicated on the graph. Data are presented as means (SD) of triplicate independent samples and experiments

Following the initial results indicating that Auraguard was involved in membrane restoration, we investigated if the ERK signalling pathway was involved in membrane restoration of infected MDCK cells. Infection of non-Auraguard treated MDCK cells with *C. jejuni* (Fig. 4A) led to ≈ 25 nmol H_2_O_2_ being secreted in the extracellular space (released H_2_O_2_) compared with ≈ 8 nmol H_2_O_2_ (p = 0.0002) in cells pre-treated with 0.25% Auraguard, which was similar to uninfected controls (Fig. 4A). Infections with *S. enterica* generated ≈ 43 nmol H_2_O_2_ which were reduced to ≈ 12 nmol H_2_O_2_ (p < 0.0001) in Auraguard pre-treated cells (Fig. 4A). Similarly, infections with *C. perfringens* (Fig. 4A) led to a decrease in H_2_O_2_ concentrations from 35 nmol H_2_O_2_ to 14 nmol H_2_O_2_ in the presence of 0.50% Auraguard (p = 0.0003). Auraguard treatment also led to significant decrease in intracellular H_2_O_2_ (p < 0.0001) (Fig. 4B). The presence of the ERK signal transduction pathway inhibitor (PD98059) also led to decrease in H_2_O_2_ both extra and intracellularly (Fig. 4A, B). The unavailability of H_2_O_2_ caused the restoration of TEER when cells were exposed to Auraguard or PD98059 (Fig. 4C), significantly decreased paracellular permeability (Fig. 4D) and restored the expression of ZO-1 (Fig. 4E). Our results also suggest that the antimicrobial mixture reduces bacterial invasion and diminishes post-infection pro-inflammatory events (Fig. 4F).

### The IL-1β, IL-6, IL-8, TNF-α levels are attenuated in infected MDCK cells by Auraguard via the ERK signal transduction pathway

**Fig. 5:**
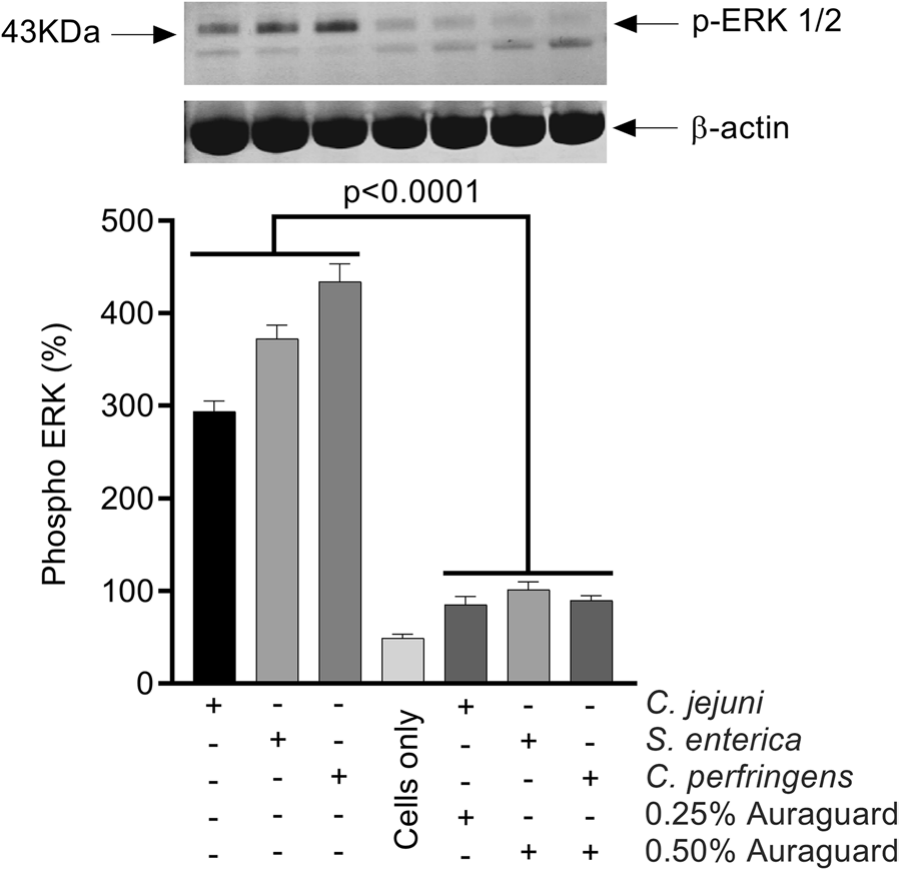
Inactivation of ERK kinases during *C. jejuni, S. enterica* and *C. perfringens* infection of MDCK cells by Auraguard. Activation of ERK was examined by Western immunoblot using monoclonal antibodies specific to the phosphorylated ERK kinase. Equal amounts of protein were added in each lane. Data were standardized on the basis of β-actin levels. The Student’s *t*-test was used to statistically compare the effect of Auraguard. p values are indicated on the graph

**Fig. 6:**
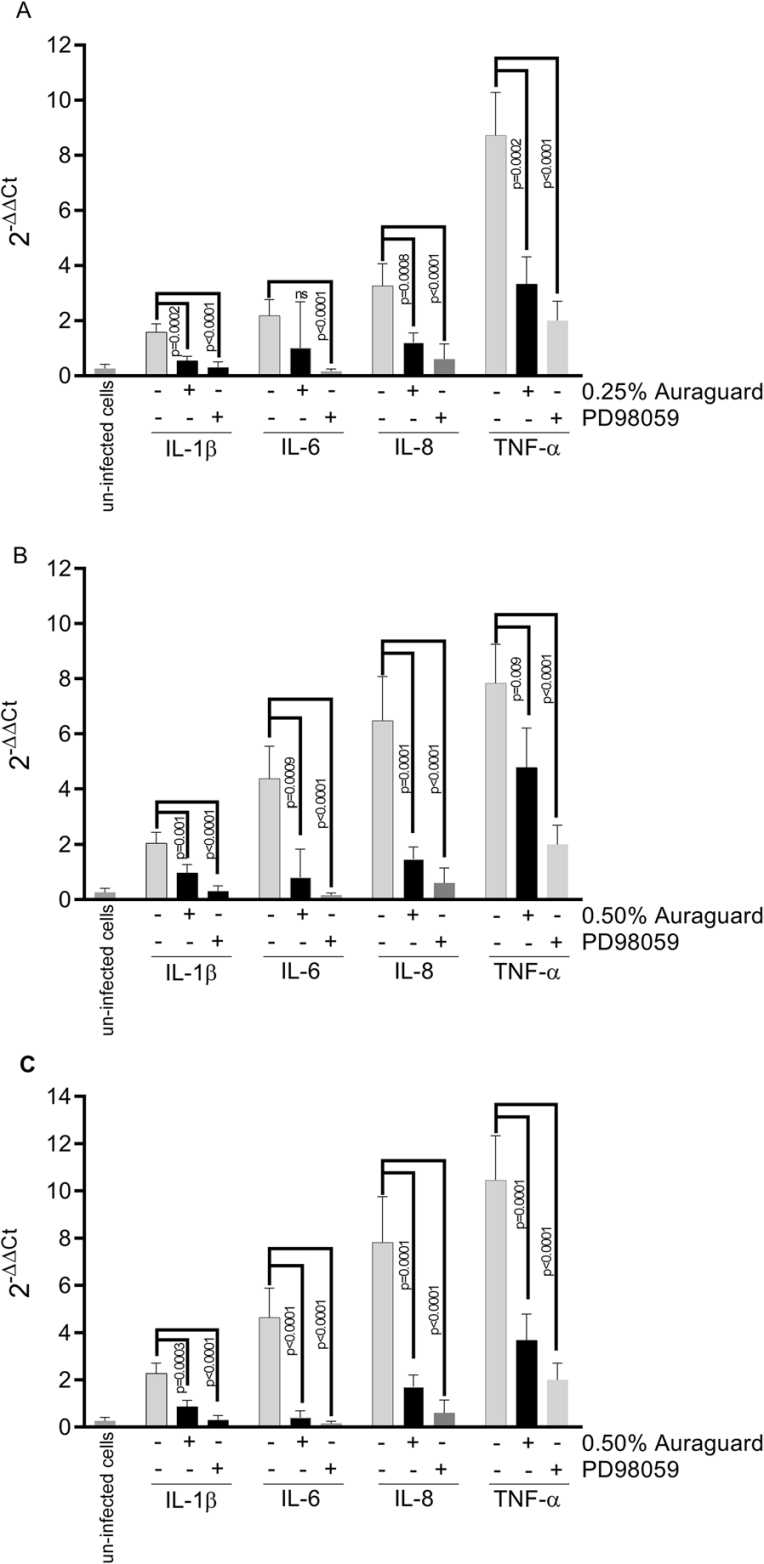
Modulation of pro-inflammatory genes expression (IL-1β, IL-6, IL-8, TNF-α) in infected MDCK cells pre-exposed to 0.25% and 0.5% Auraguard or 30 µM of PD98059. Data are expressed as 2^−DDCt^ where DCt = Ct (target gene) − Ct (housekeeping); values are the mean of three test replicates. Negative samples were given a Ct 38 fictitious value. The Student’s *t*-test was used to statistically compare the effect of Auraguard. p values are indicated on the graph

In order to further examine anti-inflammatory impacts of Auraguard we measured IL-1β, IL-6, IL-8, TNF-α secretion by infected MDCK cells. Cells were infected with *C. jejuni, S. enterica* and *C. perfringens* and ERK stimulation was assessed in cells treated at 0.25% and 0.50% Auraguard during pathogen infection using Western blot. Bacterial infection primed activation of the ERK MAP kinase, but was inhibited by Auraguard at 3 h following infection (Fig. 5). Our investigation into the role of the ERK signal transduction pathway as the mechanistic tool in reducing the inflammatory effect of bacterial invasion by Auraguard indicated that the modulation of pro-inflammatory gene expression in the MDCK cells was restored to un-infected levels by the pre-treatment with Auraguard and the ERK inhibitor PD98059. Under inflammatory conditions, there was an increased activation of IL-1β, IL-6, IL-8, TNF-α in MDCK cells following infection with *C. jejuni* RC039 (Fig. 6A), *S. enterica* (Fig. 6B) and *C. perfringens* (Fig. 6C). Cytokine levels in un-infected cells were below the detection limit (data not shown). However, our results indicate that pre-treatment with Auraguard at levels of 0.25% for *C. jejuni* (Fig. 6A) and 0.50% for *S. enterica* (Fig. 6B) and *C. perfringens* (Fig. 6C) reduced levels of IL-1β, IL-6, IL-8, TNF-α. Reduced inflammation was also observed when cells were pre-treated with the ERK inhibitor PD98059. Firstly, these results show that Auraguard reduced epithelial inflammation as indicated by the cytokine detection levels and secondly, inhibits the ERK signal transduction pathway in cultured MDCK cells. Overall, these results demonstrate that bacterial infections can activate MAP kinase pathways in cultured MDCK cells and launch pro-inflammatory events, a mechanism which is independent from bacterial internalization.

### Antimicrobial reduced inflammation results in reduction in tetrathionate production

**Fig. 7:**
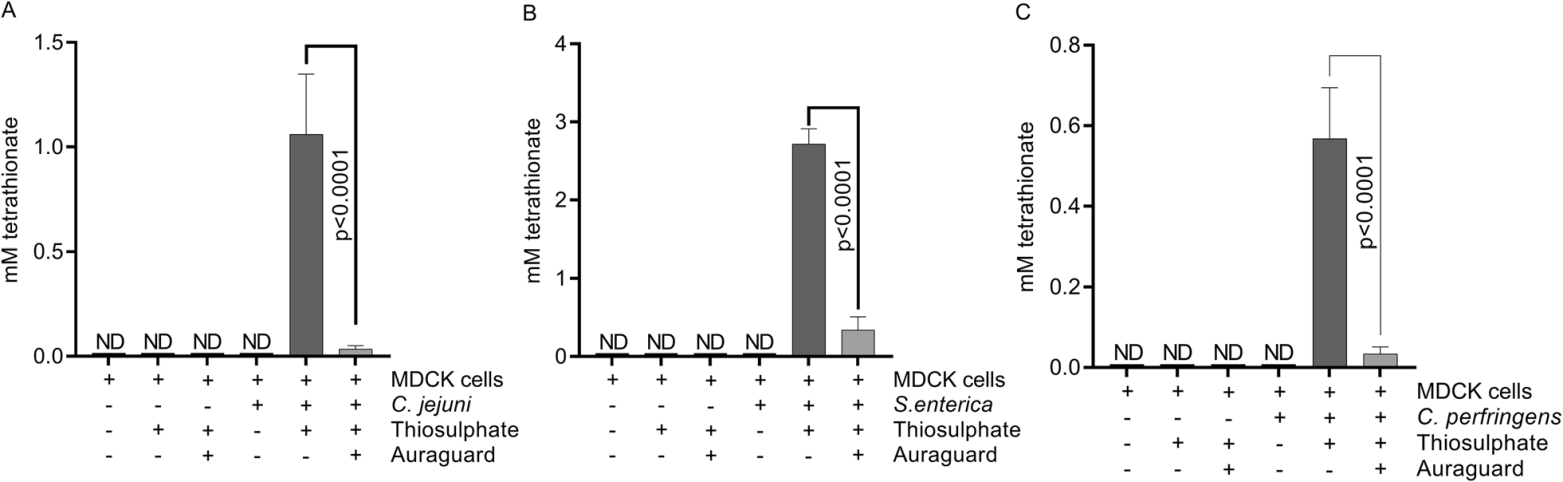
Tetrathionate levels determined by reverse phase LC–MS from supernatants of infected MDCK cells in the presence of sodium thiosulphate. **A** *C. jejuni*, **B** *S. enterica* and **C** *C. perfringens*. Data represent the mean ± SEM from three individual experiments

Treatment with the antimicrobial mixture caused a reduction in cell released reactive oxygen species (ROS) as well as inflammatory cytokine release in infected MDCK cells. This observation suggested that an impact on host driven molecules (e.g. tetrathionate) that support the growth and survival of bacterial pathogens in the gut, was also observed. The addition of 10 mM sodium thiosulfate during infection in vitro led to its conversion to tetrathionate in the supernatants of infected MDCK cells (Fig. 7). However, Auraguard inclusion decreased the levels of tetrathionate detected from 1 to 0.04 mM (p < 0.0001) in *C. jejuni* infected MDCK cell supernatants, from 2.8 to 0.3 mM in *S. enterica* infected MDCK cell supernatants (p < 0.0001) and from 0.58 to 0.025 mM in *C. perfringens* infected MDCK cells (p < 0.0001). These results indicate that in addition to the impacts of Auraguard on the extracellular and intracellular release of ROS, likely also causing a decrease in secondary molecules produced by the host during inflammation which are used as electron acceptors by bacteria in oxygen depleted environments.

## Discussion

Understanding the biological mechanisms of how natural antimicrobials prevent infection and subsequent inflammation is limited. In certain pathogens (e.g. *Campylobacter jejuni*) the mechanism of epithelial cell invasion is mechanistically separated from cellular inflammation [22], involving the extracellular signal transduction pathway (ERK) as an activation switch. ERKs are activated by bacteria through a mechanism involving posttranslational modifications through tyrosine phosphorylation leading to pro-inflammatory cytokine production [28]. Natural antimicrobials have previously been shown to have the ability to block ERK activation and prevent bacterial-induced inflammation [25].

Bacterial pathogens including *C. jejuni, S. enterica* and *C. perfringens* are known for their ability to disrupt epithelial barriers and stimulate epithelial production and release of H_2_O_2_ in both the intracellular and extracellular spaces [20, 29]. Infection of MDCK cells with *C. jejuni, S. enterica* or *C. perfringens* generated an increase in H_2_O_2_ detected in the intracellular and extracellular spaces. In MDCK cells the increase in H_2_O_2_ production was associated with enhanced paracellular permeability involving the disruption of ZO-1 protein within the actin cytoskeleton [30, 31]. It has been suggested that the molecular mechanisms controlling and modulating the paracellular permeability required the involvement of occludin and ZO-1 proteins [32, 33]. Inclusion of Auraguard in all infections by *C. jejuni*, *S. enterica* and *C. perfringens* reduced membrane permeability, increased ZO-1 expression in MDCK cells and reduced H_2_O_2_ production. All these events were followed by reduced invasive abilities of *C. jejuni*,* S. enterica* and *C. perfringens* in these in vitro cell lines. Organic acids (e.g. citrus extracts) have been shown to reduce the generation of reactive oxygen species thereby preventing oxidative cell injury [34] and when used in combinations will restore TEER and improve cellular tight junctions [26]. The Auraguard-catalysed reduction in H_2_O_2_ released by the infected MDCK cells was linked to the observed increased expression of the ZO-1 protein. The higher levels of H_2_O_2_ reduced occludin and ZO-1 protein content and their involvement in regulating the paracellular permeability [35, 36].

It remains unknown how tight junction structures are affected by endogenous H_2_O_2_ production and how natural antimicrobials (e.g. organic acids) impact these structures. Extracellular signal-regulated kinases (ERKs) mediate tight junction disruption, and this mediation takes place in the cytoplasm being restrictively activated by H_2_O_2_ [21, 37]. Secondly, from a prokaryotic perspective, H_2_O_2_ activates the ERK signal transduction pathway triggering production of pro-inflammatory cytokines in infected intestinal cells [22, 38–40]. Thirdly, organic acids (e.g. Ambuic acid) exert anti-inflammatory effects by blocking the ERK signalling pathway [41]. In this study Auraguard blocked the H_2_O_2_ activated ERK signalling pathway, prevented inflammation and averted the damaging effect on tight junction structures. However, inhibition of the ERK signalling pathway did not prevent invasion of MDCK cells by *C. jejuni, S. enterica* and *C. perfringens* suggesting that the anti-inflammatory mechanism of Auraguard was disconnected from the anti-invasive mechanism. Our study clearly demonstrates that the phospho-deactivation of ERK by Auraguard did not stop bacterial invasion, but did reduce subsequent inflammation. Decoupling of invasion from inflammation was previously described by *Campylobacter jejuni* where epithelial cell invasion was not necessary to initiate the pro-inflammatory cascade [22]. Reduction in inflammatory cytokines was associated with increased TEER values, ZO-1 expression, and occludin expression suggesting that these cytokines could modulate tight junction integrity and reduce cellular permeability. Specifically, it has been suggested that downstream effectors of MAP kinase signalling pathways mediate a reorganization of cellular barrier function via TNF-α [42]. The cytokine mediation likely coordinates the functions of caveolin-1, involved in cholesterol-rich membrane microdomains [32].

Inflammation of the host epithelium enhances the ability of some bacterial pathogens to grow and survive. *Salmonella*, similar to other pathogens [20], triggers the production of reactive oxygen species by neutrophils which oxidise thiosulphate into tetrathionate, a product used by *Salmonella Typhimurium* [43], *Yersinia enterocolitica* [44] and *Campylobacter jejuni* [45] to grow in anaerobic conditions. In vivo thiosulphate is produced through oxidation of H_2_S by the colonic epithelium and further oxidised tetrathionate by commensal bacteria. Along with our observation of the reduction in intra and extracellular H_2_O_2_ release, we also detected a reduction in tetrathionate production as a result of Auraguard-catalyzed inhibition of H_2_O_2_ production. Our results indicate, that in vivo, Auraguard could potentially reduce epithelial inflammation and also prevent the supply of host molecules (e.g. tetrathionate) that could promote the growth and survival of bacterial pathogens in oxygen-depleted environments.

## Conclusion

In previous studies we have shown that mixtures of natural antimicrobials are able to reduce bacterial virulence and improve the host defence mechanisms using both in vitro and in vivo infection models [11–13, 15, 16, 46]. The current study elucidates the biological mechanism that underlies the anti-pathogenic and anti-inflammatory effects of Auraguard, a mixture of natural antimicrobials. We found that this type of natural antimicrobial mixtures inhibited bacterial internalisation through the restoration of epithelial structures damaged by invasion. Secondly, they act via an anti-inflammatory mechanism involving the deactivation of the ERK signalling pathway through de-phosphorylation. We also show that these mechanisms are similar across pathogenic species as the effects were observed in the case of *C. jejuni*, *S. enterica* and *C. perfringens* infections. Possible synergistic mechanisms and interactions between individual components indicate the need for further in vivo investigations are needed to extrapolate these results to animal disease models and possibly to compare their effects to those of various antibiotics.

## Material and methods

### Bacterial strains and the mixture of natural antimicrobials

*Campylobacter jejuni* RC039 [47], *Salmonella enterica* serovar Typhimurium SE10/72 [48], including *Clostridium perfringens* ATCC® 13124™ [49] strains, were obtained from the AFBI laboratory collection. *C. jejuni* RC039 was grown on Blood Agar Base No. 2 (Oxoid Ltd., United Kingdom) supplemented with 5% (vol/vol) defibrinated horse blood (Aquilant Scientific N.I.). *S. Typhimurium* SE10/72 was resuscitated by adding one bead to tryptone soy broth (Oxoid, Basingstoke, UK) plus 0·6% (w/v) yeast extract (Oxoid) and incubating at 37 °C for 24 h. Working cultures were maintained on TSAYE slants at 4 °C. Working cultures were prepared by inoculation of MHB with a loopful of culture from a slope and incubating at 37 °C, for another 24 h. *C. perfringens* ATCC® 13124™ was similarly resuscitated into anoxic Müeller Hinton broth (MHB) and then incubated anaerobically at 37 °C for 24 h. Working cultures were maintained on cooked meat medium at 4 °C for 30 days. Working cultures were prepared by inoculation of MHB with 20 μl of overnight culture grown anaerobically at 37 °C in a cooked meat medium. To create anaerobic conditions, Anaerogen™ (Thermo Scientific™ Oxoid™) sachets were added to the 2.5 litter jar specified to maintain anaerobiosis. Thorough all experimental procedures, the *C. perfringens* ATCC® 13124™ strain was maintained and grown under anoxic environmental condition. The antimicrobial mixture, Auraguard, contained: 5% maltodextrin, 1% sodium chloride, 42% citric acid, 18% sodium citrate, 10% silica, 12% malic acid, 9% citrus extract and 3% olive extract (w/w). The raw materials were supplied by Bioscience Nutrition Ireland.

### Disc diffusion assay

Briefly, 100 μl of *S. Typhimurium* and *C. perfringens* bacterial cultures were spread onto Tryptone Soya Agar plus yeast extract (TSAYE Oxoid, UK), and *C. jejuni* was plated on Blood Agar Base Nr. 2 (BAB, Oxoid Ltd., United Kingdom) enriched with 5% (vol/vol) defibrinated horse blood (Aquilant Scientific N.I.). Next, the antimicrobial product was diluted in tenfold increments from 100% to 0.078% (v/v) in MHB and 20 μl of corresponding antimicrobial concentration was absorbed on 6 mm paper discs (Oxoid, UK). Antimicrobial activity of each antimicrobial was evaluated by the disc diffusion method according to the measuring of the diameter of the inhibition zone around the disc for each of antimicrobial concentrations used.

### Determination of minimum inhibitory and minimum bactericidal concentration

The two-fold tube dilution method was used to determine the lowest concentration of Auraguard that inhibited bacterial growth (MIC) and the lowest concentration that induced bacterial death (MBC) was evaluated [50]. Auraguard was diluted (8% to 0.015625% v/v) in Müeller Hinton broth (MHB) and thoroughly vortexed. Individual overnight bacterial cultures were harvested by centrifugation, washed twice in PBS, resuspended in MHB, and diluted to 1 × 10^6^ CFU/ml in MHB. Each tube was inoculated with 5 × 10^5^ CFU/ml of each bacterial culture (final concentration). Non-inoculated bijou (5 ml) tubes containing the same growth medium were used as negative controls, whilst MHB tubes without Auraguard were inoculated with individual bacterial cultures as positive controls. Subsequently, *C. jejuni* tubes were incubated at 41.5 °C for 48 h, and *C. perfringens* and *S. Typhimurium* cultures were incubated at 37 °C for 24 h. Tubes that did not show visible growth were considered to be above the MIC. One hundred millilitres were taken from each tube for inoculation and then incubated at 37 °C for 24 h onto TSAYE on Campylobacter Blood-Free Selective Agar Base (Modified CCDA—Preston; Oxoid Ltd., United Kingdom) under microaerophilic at 41.5 °C for 48 h. The highest dilution of each antimicrobial with no microbial growth was considered as the MBC [51]. The antimicrobial mixture was tested using concentrations from 8 to 0.0078% (v/v) in three independent replicates repeated three times for each strain. In order to determine the sub-inhibitory concentrations used, all three pathogens were exposed to different concentrations of the antimicrobial mixture. The highest concentrations of antimicrobial that showed no effect on survivability and no growth inhibition (same growth kinetics as the control) were used in subsequent experiments [15].

### Infectivity assays

The ECACC Madin–Darby Canine Kidney cells (MDCK) were grown in DMEM (Lonza, Analab Ltd., United Kingdom) supplemented with 10% foetal bovine serum. The cells were routinely grown in 75cm^2^ tissue culture flasks (Sigma-Aldrich, Arklow, Ireland, SIAL0641) in a humidified incubator at 37 °C with 5% CO2. The gentamicin protection assay was used to test the role of Auraguard towards the ability of *C. jejuni* RC039, *S. Typhimurium* SE 10/72 and *C. perfringens* ATCC® 13124™ to adhere to and invade host epithelial cells as previously explained [52]. MDCK cells were grown (80–90% confluence) for 22–24 h in six-well tissue culture plates at a concentration of 5.5 × 10^5^ cells per well. The MDCK cell monolayers were pre-incubated with 0.25 and 0.5% Auraguard for 2–3 h. The pH during all the experimental infections studies was maintained at neutral values (pH 7.2). Plate grown cultures of *C. jejuni* RC039, *S. Typhimurium* SE 10/72 and *C. perfringens* ATCC® 13124™ were harvested, washed, and re-suspended in tissue culture medium at an OD600 of ≈ 0.3. The MDCK cells were washed twice with fresh culture media supplemented with 10% FBS, and 2 ml of fresh culture medium was added to each well. Bacteria were added to give a multiplicity of infection of 100. Then, tissue culture plates were centrifuged at 250×*g* for 5 min, and the plates with *Campylobacter* were incubated for 3 h at 41.5 °C in 85% N_2_, 5% O_2_ and 10% CO_2_. Simultaneously, the six-well plates with *Salmonella* and *Clostridia* added for infection assays were subjected to 37 °C incubation for 2 h. To quantify the number of cell-associated bacteria, infected monolayers were washed three times with PBS and treated with 0.1% Triton X-100 in PBS at 41.5 °C and 37 °C for 15 min. Ten-fold dilutions from each well were plated onto CCDA, and TSAYE agar (depending on bacteria culture) and the colonies were enumerated after 2 days of incubation at 41.5 °C in 85% N_2_, 5% O_2_, and 10% CO_2_ or at 37 °C respectively. In order to inhibit the ERK signal transduction pathway 30 µM of PD98059 (Sigma-Aldrich, UK) was added to the infection plate 60 min prior to infection. The infected monolayers were washed with tissue culture medium to quantify the number of bacteria that invaded MDCK cells. Fresh medium (2 ml) containing gentamicin (400 μg/ml) was added to kill bacteria that were not internalized. Medium without gentamicin was introduced to quantify the number of bacteria that adhered to the epithelial cells. Next, the tissue culture plates were incubated for a further 3 h at 41.5 °C or 37 °C and washed with fresh DMEM + 10% FBS. MDCK cells were lysed by the addition of 1 ml of 0.1% Triton X-100 in PBS and incubated for 15 min at 41.5 °C or 37 °C. Tenfold dilution of each well content was plated onto CCDA and TSAYE agar, and colonies were enumerated after 1–2 days of incubation. Invasion efficiency was calculated as the percentage of the total number of CFU/total initial inoculum. All assays were conducted in triplicate on three separate days. The significance of differences in adhesion and invasion between samples was determined using the Student’s t-test. A p-value of < 0.05 was defined as significant.

### Transepithelial electrical resistance (TEER) and paracellular permeability measurements

MDCK cells were plated onto transwells (5 × 10^4^; 6.5 mm diameter; 0.4 μm—pore size; Corning) and grown until apical junctional complexes developed. Transwells were infected apically with either *C. jejuni* RC039, *S. enterica* SE 10/72 or *C. perfringens* ATCC® 13124™. TEER was measured at 3 h after infection using an EVOM X meter connected to an Endohm chamber (World Precision Instruments). The paracellular permeability of MDCK cells was determined using calcein as the solute as described previously [53]. Flux assay data are presented as means (SD) of triplicate independent samples performed three separate times. The presented results are representative of at least three independent experiments. The mean (SD) of at least three independent experiments for each cell line was calculated.

### Gene expression analysis

The quantification of zonula occludens-1 (ZO-1) and occluding expression was performed as previously described with small modifications [26]. Briefly, the exposed or infected MDCK cells were snap-frozen in liquid nitrogen until use. RNA was isolated using RNeasy Plus Mini Kit (Qiagen, United Kingdom). The RNA was reverse transcribed using Transcriptor First Strand cDNA Synthesis Kit (Roche) according to the manufacturer’s protocol. The mRNA levels were determined by quantitative RT-PCR using QuantiNovaSYBR Green PCR Kit (Qiagen, United Kingdom) on a LightCycler 96 (Roche). The primers used for ZO-1 were F: CGGGACTGTTGGTATTGGCTAGA and R: GGCCAGGGCCATAGTAAAGTTTG. For the occluding gene the primers used were F: TCCTATAAATCCACGCCGGTTC and R: CTCAAAGTTACCACCGCTGCTG. The gene expression was normalized using the ribosomal protein lateral stalk subunit P0 (RPLP0) and glyceraldehyde-3-phosphate dehydrogenase (GAPDH). The 2–DDCT method was used to analyse the relative expression (fold changes), calculated relative to the control group. In order to test the anti-inflammatory after LPS or Auraguard treatment we evaluated the expression of the following genes: IL-1β, IL-6, IL-8, and TNF-α. The expression of GAPDH gene was used as a control. The primers used were: for IL1-β F-TGCAAAACAGATGCGGATAA, and R-GTAACTTGCAGTCCACCGATT; IL-6, F-TCCAGAACAACTATGAGGGTGA, R-TCCTGATTCTTTACCTTGCTCTT; IL-8, F-TGATTGACAGTGGCCCACATTGTG, R-GTCCAGGCACACCTCATTTC; TNF-α, F-CGTCCATTCTTGCCCAAAC, R-AGCCCTGAGCCCTTAATTC and for GADPH, F-TTCCACGGCACAGTCAAG, R-ACTCAGCACCAGCATCAC. The relative expression of the selected genes was calculated using the formula 2-DDCt with DCt values being represented as means of three test replicates [54].

### Intra and extracellular hydrogen peroxide (H_2_O_2_) measurements in infected MDCK cells

Hydrogen peroxide (H_2_O_2_) production was measured using a Hydrogen Peroxide Detection Kit (Enzo Life Sciences) or Amplex® UltraRed /HRP (Thermo Fischer Scientific, UK) according to the manufacturers’ instructions. Briefly, the lysis buffer or culture media (50 ml) were mixed with the Amplex® UltraRed /HRP (Thermo Fischer Scientific, UK) reagent and with the horseradish peroxidase resulting in a red fluorescent oxidation product. Fluorescence was determined at 530 nm excitation and 590 nm emission using a fluorescence microplate reader (FLUOstar Omega, BMG Labtech). The concentrations of H_2_O_2_ were calculated using standard curves. For intracellular measurements, after treatment with Auraguard the confluent cells were washed with phenol red free DMEM and incubated in the dark for 10 min in Krebs–Ringer solution containing 2′,7′-dichlorofluorescin diacetate (DCF-DA; Sigma Aldrich, UK). Cell cultures treated with diatrizoate (6%) or ioxag- late (10%) were detached from culture flasks, counted (1 × 10^6^ cells), and re-suspended in 800 μl of PBS. A solution of DCFH (200 μl) at final concentration 20 μmol/ml were added to the samples and incubated with agitation at 37 °C for 30 min. The cells were centrifuged, resus- pended in EDTA, and carried out for fluorescence activated cell sorting (FACS) analysis. The values were averaged to obtain the mean relative fluorescence intensity, and the means were compared with each well. All experiments were repeated three times.

### Western blotting

The infected cells, treated or un-treated with Auraguard, were boiled with SDS‑PAGE loading buffer. Protein samples (40 μg) were subjected to SDS-PAGE and further transferred on nitrocellulose membranes. The membranes were blocked with 5% dried milk in Tris-buffered saline and Tween-20 (TBST, 20 mM Tris‑HCl, 150 mM NaCl, 0.05% Tween-20) for 6 h at room temperature. After washed with TBST, incubating the membranes in respective primary antibody solution (anti‑phospho‑ERK and anti‑β‑actin antibody, from Santa Cruz Biotechnology) overnight at 4˚C. After extensive washing, the membranes were then incubated with HRP‑conjugated secondary antibody solution for 1 h at room temperature. The membranes were washed three times with TBST and the blots were detected by using enhanced chemiluminescence reagent (ECL) and exposed to photographic films (Kodak, Thermo Fischer, UK). Images were collected and the bands corresponding to phospho‑ERK protein were quantitated by densitometric analysis using the E-Gel Imager from Life Technologies. Data of phospho‑ERK were normalized on the basis of β‑actin levels.

### Measurement of tetrathionate production

MDCK cells were plated onto transwells (5 × 10^4^; 6.5 mm diameter; 0.4 –pore size; Corning). Transwells were infected apically with either *C. jejuni* RC039, *S. enterica* SE 10/72 or *C. perfringens* ATCC® 13124™. The cells were infected as described above. Sodium thiosulfate was added in each infection transwell, 60 min after infection, at a concentration of 10 mM. For measurements of tetrathionate produced by MDCK cells infected or infected in the presence of Auraguard (0.25 for *C. jejuni* and 0.50% for *S. enterica* and *C. perfringens*) Dimethylsulfoxide or Phorbol 12-myristate 13-acetate (in Dimethylsulfoxide) was added to tissue culture media at a final concentration of 1% and 10 µg/ml, respectively, and the cells were incubated for 3 h at 37 °C 5% CO_2_. All samples were filter sterilized, and analysed using ion pairing RP-LC-MS as previously described [44, 55].

### Statistical analysis

Statistical analyses were performed using GraphPad software. Data were represented as mean ± SD. p-values < 0.05 were considered statistically significant following estimations using the Student t was used.

## Data Availability

All data generated or analysed during this study are included in this published article.

## Abbreviations

PCR: Polymerase chain reaction
DNA: Deoxyribonucleic acid
MDBK: Madin–Darby bovine kidney cells
PBS: Phosphate buffer saline
GIT: Gastrointestinal tract
